# Impact of Short-Term Hypoxia on Sirtuins as Regulatory Elements in HUVECs

**DOI:** 10.3390/jcm9082604

**Published:** 2020-08-11

**Authors:** Simone Johanna Pecher, Arne Björn Potthast, Frauke von Versen-Höynck, Anibh Martin Das

**Affiliations:** 1 Clinic for Pediatric Kidney, Liver and Metabolic Diseases, Hannover Medical School (MHH), Carl-Neuberg-Straße 1, 30625 Hannover, Germany; simonepecher@gmx.de (S.J.P.); arne_potthast@gmx.de (A.B.P.); 2 Department of Obstetrics and Gynecology, Hannover Medical School (MHH), Carl-Neuberg-Straße 1, 30625 Hannover, Germany; vonversen-hoeynck.frauke@mh-hannover.de

**Keywords:** sirtuins, SIRT1, SIRT3, SIRT4, HUVECs, endothelial metabolism, NAD^+^, hypoxia, ROS, β-oxidation

## Abstract

Background: Sirtuins (SIRT) are NAD^+^-dependent deacetylases that are involved in stress response, antioxidative defense, and longevity via posttranslational modifications. SIRT1 directly activates nitric oxide synthase (NOS). Aging is associated with a reduced sirtuin function and reduction of the cofactor NAD^+^. Age-related atherosclerosis and vascular diseases are linked to a compromised sirtuin function. Vascular events like stroke and cardiac infarction result in acute hypoxia, which can additionally impact sirtuins and thus the vascular function. This prompted us to study sirtuins in intact HUVECs, under acute, short-term hypoxic conditions. Methods: We measured intracellular sirtuin and NAD^+^ levels in HUVECs exposed to hypoxia (2% O₂) for 10–120 min, compared to normoxic controls. SIRT1, SIRT3, and SIRT4 were measured at the protein (Western Blot) and the transcript level (qRT-PCR), SIRT1 and SIRT3 at the enzyme level (fluorometrically), and NAD^+^ levels were measured spectrophotometrically. Results: We observed a reduction of SIRT1 and SIRT4 at the protein level, a downregulation of SIRT1 at the transcript level and increased NAD^+^ levels under hypoxia. SIRT3 was not affected by hypoxia. Conclusions: Downregulation of SIRT1 under hypoxia might reduce production of the reactive oxygen species (ROS) via the respiratory chain and inhibit the mitochondrial ATP-synthase, resulting in energy conservation. NOS might be impaired if SIRT1 is decreased. Increased NAD^+^ levels might compensate these effects. Hypoxic downregulation of SIRT4 might lead to mitochondrial uncoupling, hence endothelial dysfunction, and ADP/ATP-translocase 2 (ANT2)-inhibition. NAD^+^ upregulation might partly compensate this effect.

## 1. Introduction

In recent years, sirtuins (SIRTs) were shown to regulate vascular and especially endothelial functions under physiological conditions [1,2,3,4].

Sirtuins are nicotinamide adenine dinucleotide (NAD^+^)-dependent deac(et)ylases [5,6], first discovered in saccharomyces cerevisiae as silent mating-type information regulator 2 (Sir2) [7], and later observed in mammalian cells. In humans, seven different forms (SIRT1-7) are known [8], they are localized in different cellular compartments—SIRT1, primarily detected in the nucleus, is able to shift to the cytosol. SIRT2 is found in the cytosol, SIRT1, 6 and 7 in the nucleus and SIRT3, 4 and 5 in mitochondria [9]. For enzymatic activity, all sirtuins require oxidized NAD^+^ as an essential cofactor/substrate [10]. The most common enzymatic sirtuin activity is deacetylation of acyl-lysine residues, catalyzed by SIRTs 1, 2, 3, 4, 6, and 7. Through this reaction, the side products *O*-acetyl-ADP-ribose and nicotinamide (NAM) are formed [11]. SIRT1, SIRT4, and SIRT6 catalyze ADP-ribosylation as well [12].

As NAD^+^-dependent deacetylases, sirtuins are directly linked to cellular energy metabolism. Via the NAD^+^/NADH-system, sirtuin pathways are linked to the mitochondrial respiratory chain (RC), which is blocked under hypoxia, resulting in a shift from NAD^+^ to NADH. If the essential cofactor NAD^+^ is not available, sirtuin activity is restricted. Poly-ADP-ribose polymerases (PARPs) [13] compete with sirtuins for the same intracellular NAD^+^ pool. Activated by DNA damage, PARPs degrade NAD^+^, and sirtuin activity decreases [14]. Sirtuins convert NAD^+^ to NAM. In higher concentrations, NAM non-competitively binds the sirtuins and inhibits feedback [15].

Intact endothelial cells prevent vascular events by anti-platelet, anti-coagulant, fibrinolytic, anti-inflammatory, and antiproliferative effects [16]. Endothelial cells produce vasodilators such as nitric oxide (NO), as well as vasoconstrictors, for example, endothelin-1. Previously, it was reported that SIRT1 can deacetylate and activate NO-synthase (NOS) [17,18,19].

Under pathophysiological conditions like cardiovascular disease [20,21,22,23,24] and vascular aging including atherosclerosis [1,25,26,27,28], altered functions of sirtuins were observed. Increasing age is associated with lower sirtuin activity [29,30,31], making older people more susceptible to vascular disease. This is partly due to the reduced levels of the sirtuin cofactor NAD^+^ (for a review see Das, and Dabke [32], in press). Activation of SIRT1 was shown to have a protective effect on the process of atherosclerosis [33,34].

Acute hypoxia is a condition underlying different clinical pathologies. This might occur as pure hypoxia, for example, in lung injury or anemia following hemorrhage. Hypoxia might be either generalized or regional. Ischemia, i.e., lack of blood flow, on the other hand, is mostly regional and always accompanied by hypoxia [19]. Examples for hypoxic events are perinatal complications (such as pre-eclampsia and the HELLP syndrome), which might not only result in hypoxia within the vessel wall [35] but also in placental ischemia [36]. Transplantation of solid organs and major surgery are other conditions associated with hypoxia of organ tissues.

Atherosclerosis is the most common vascular compromise associated with aging. The condition might lead to acute ischemia in the context of embolic or thrombotic processes clinically manifesting as stroke or cardiac infarction. Acute hypoxia in the context of stroke or cardiac infarction might have an additional effect (2nd hit) on sirtuins in atherosclerotic endothelial cells. 

Hypoxia is associated with cellular energy deficiency and production of reactive oxygen species (ROS), which promote oxidative damage of cellular macromolecules [37], and might cause disruption of calcium homeostasis [38].

We hypothesized that acute, short-term hypoxia triggers alterations in the sirtuin function, either directly or indirectly via the cofactor NAD^+^, in intact HUVECs, thus, having an impact on the endothelial function and the clinical outcome. In the present study, we focused on alterations of three sirtuins, i.e., SIRT1, SIRT3, and SIRT4, as the regulatory elements in energy metabolism and other physiological processes in vascular endothelial cells, under acute, short-term hypoxic conditions. The biological functions of these sirtuins are briefly mentioned below.

The first described function of sirtuin 1 (SIRT1; EC: 3.5.1.17) was histone deacetylation [6]. SIRT1 regulates metabolic processes via the transcription factor peroxisome proliferator-activated receptor α (PPARα), with subsequent activation of fatty acid oxidation [39,40]. SIRT1 activates the peroxisome proliferator-activated receptor-γ co-activator 1α (PGC-1α) and regulates mitochondrial biogenesis, oxidative phosphorylation, and glucose homeostasis, via an adenosine monophosphate-activated protein kinase (AMPK)-dependent signaling pathway [41,42,43]. By activating the forkhead box protein O 3 (FOXO3) transcription factor, SIRT1 has an antioxidative effect [44]. Vascular endothelial cells are directly regulated by SIRT1 via deacetylation of the endothelial NOS (eNOS), thus, increasing its activity and vascular relaxation [45]. During senescence, SIRT1 expression is downregulated [46], which presumably leads to an increased risk of age-related diseases [47].

Sirtuin 3 (SIRT3; EC: 3.5.1.17) impacts mitochondrial metabolism. A proteomic investigation showed that more than one-third of all mitochondrial proteins have acetylation sites [48], which make them amenable to regulation by sirtuins [49,50,51]. SIRT3 enhances the resistance to various mitochondrial stressors [52], such as caloric restriction (CR) and exercise [53,54], and also regulates fatty acid oxidation by inducing long chain acyl coenzyme A dehydrogenase (LCAD) [55]. Furthermore, SIRT3 regulates the respiratory chain complexes NDUFA9 [56], succinate dehydrogenase subunit A (sdhA) of complex II [57], complexes III and IV [58], as well as ATP synthase [59]. Since ROS are formed in the respiratory chain, antioxidative protection is necessary [60]. This is mediated by SIRT3 via superoxide dismutase 2 (SOD2), isocitrate dehydrogenase (IDH2), glutathione reductase (GR), and FOXO3 [49,53,61]. SIRT3 regulates the mitochondrial permeability transition pore (mPTP) in cardiomyocytes [62].

Sirtuin 4 (SIRT4; EC: 2.4.2.30) regulates ATP homeostasis via AMPK and ADP/ATP-translocase 2 (ANT2) [63]. SIRT4 inhibits malonyl-CoA decarboxylase thus leading to increased malonyl-CoA levels [64]. Long-chain fatty acid transport to the mitochondrial matrix is inhibited by malonyl-CoA, which blocks carnitine palmitoyltransferase 1 (CPT1).

In the present study, we focus on alterations of sirtuins as regulatory elements of energy metabolism and other physiological processes in vascular endothelial cells under acute, short-term hypoxic conditions. We hypothesize that sirtuins are altered by acute hypoxia in endothelial cells.

## 2. Experimental Section

### 2.1. Cell Culture

Primary human umbilical vein endothelial cells (HUVECs) were isolated from umbilical cords from uncomplicated term-pregnancies after vaginal delivery, and were cultured at 37 °C and 5% CO₂ in 75 cm² polystyrene flasks (Sarstedt AG & Co. KG, Nümbrecht, Germany) [65]. Using flow cytometry, the typical endothelial cell phenotype (CD31+, CD90-) was confirmed [66,67]. We used Endothelial Cell Growth Medium with Supplement Mix (Promo Cell GmbH, Heidelberg, Germany) containing 2% (v/v) fetal bovine serum (PAA Laboratories GmbH, Pasching, Austria) and 1% (v/v) penicillin/streptomycin (PAA Laboratories GmbH, Pasching, Austria). HUVECs from passages 4–6 were used for experimentation. Written informed consent from the parents/mothers was obtained, before they participated in the study. The study protocol for isolating HUVECs was conducted in accordance with the Declaration of Helsinki, and was approved by the local ethics review board (project identification code 3998).

The median age of the study participants was 32 years. All women were healthy Caucasians, without pre-existing hypertension, diabetes, or other diseases affecting the endothelial function, with a median pre-pregnancy body mass index (BMI) of 21.1 kg/m². They had singleton, uneventful pregnancies and spontaneous vaginal deliveries (two boys and one girl), with a median arterial cord blood pH of 7.38 and a median infant birth weight of 3460 g.

### 2.2. Hypoxia and Normoxia Conditions

At 80 to 95% confluence, the cells were exposed to 2% O₂ for 10, 60, and 120 min at 37 °C in an Xvivo v0.9.3 incubator (BioSpherix, Ltd., Parish, NY, USA). We chose these relatively short incubation times to simulate acute hypoxia in the clinical setting, which is generally kept as short as possible. For example, interventional therapy in stroke or cardiac infarction is performed as soon as possible, after clinical onset of symptoms. Perinatal hypoxia, if occurs, is typically a short event. Surgery and transplantation are also associated with hypoxia, which is kept as short as possible. The choice of the period of the short-term hypoxia was based on studies already conducted by other groups, such as Brunssen et al. (short-term hypoxia 60–240 min) and Wein et al. (short-term hypoxia 30–60 min) [68,69].

Cell lysis was performed under hypoxic conditions. All hypoxic cells were compared to the control cells from the same cell line, cultured in parallel, under normoxic conditions (21% O₂). We used three different cell lines in three biological replicates. Measurements were done in triplicates, both for hypoxia and normoxia (controls).

### 2.3. RNA Isolation

Cells were diluted in 350 µL buffer RLT (Qiagen GmbH, Hilden, Germany) containing 1% (v/v) ß-mercaptoethanol and isolated with RNeasy Micro Kit, QIAshredder, and RNAse free DNase Set (Qiagen GmbH, Hilden, Germany), following the manufacturer’s protocol.

### 2.4. cDNA Synthesis

A total of 1000 ng of total RNA from each sample were used for the cDNA synthesis by processing with the Omniscript RT Kit (Qiagen GmbH, Hilden, Germany) according to the manufacturer’s protocol.

### 2.5. Quantitative Real-Time PCR for mRNA Expression Analysis

Real-time PCR of cDNA samples was carried out with SYBR green on 7300 Real Time PCR System (Applied Biosystems, Fisher Scientific GmbH, Schwerte, Germany). The following human primers were used for the analysis: β-actin (forward): TTC CTG GGC ATG GAG TC; β-actin (reverse): CAG GTC TTT GCG GAT GTC; SIRT1 (forward): CAA CTT GTA CGA AGA C; SIRT1 (reverse): TCA CCG AAC AGA AGG; SIRT3 (forward): CAG TCT GCC AAA GAC CCT TC; SIRT3 (reverse): AAA TCA ACC ACA TGC AGC AA; SIRT4 (forward): GCT GTG AGA GAA TGA AGA TGA GC; SIRT4 (reverse): CTT GGA AAG GGT GAT GAA GCG; MnSOD (forward): GTG GAG AAC CCA AAG GGG AG; MnSOD (reverse): GCC TGT TCC TTG CAG TG. β-Actin was used as the internal control. Relative changes in the mRNA expression were calculated according to Livak et al. [70].

### 2.6. Western Blot Analysis

A total of 50,000 cells lysed in 3x Laemmli’s loading buffer were separated by SDS-Page (10% separation gel and 4% collection gel), transferred to nitrocellulose membranes by semi-dry blotting, blocked with 5% blocking milk solution or Odyssey Buffer with 5% BSA, for one hour at room temperature, and incubated overnight with specific primary antibodies at 4 °C. The blots were incubated with secondary antibodies, for one hour at room temperature, membranes were developed either by X-ray Film Processor (Protec GmbH & Co. KG, Oberstenfeld, Germany) through enhanced chemiluminescence or Li-Cor Odyssey Fc by fluorescent antibodies (Odyssey IRDye, LI-COR Biosciences GmbH, Bad Homburg, Germany).

Primary antibodies used were SIRT1 rabbit monoclonal (MerckMillipore, Merck KGaA, Darmstadt, Germany) (MW: ~130 kDa), SIRT3 rabbit polyclonal (MerckMillipore, Merck KGaA, Darmstadt, Germany) (MW: ~46 kDa); SIRT4 rabbit polyclonal (Santa Cruz Biotechnolgy, Inc., Dallas, USA) (MW: ~39 kDa); α-Tubulin rabbit polyclonal (Cell Signaling Technology, Inc., Danvers, USA) (MW: ~52 kDa).

HRP-conjugated secondary antibodies were bovine anti-mouse IgG; goat anti-rabbit IgG (Santa Cruz, USA). We used fluorescent antibodies for detection with Li-Cor Odyssey Fc Goat anti-mouse Odyssey IRDye 800CW; goat anti-rabbit Odyssey IRDye 680CW (LI-COR Biosciences GmbH, Bad Homburg, Germany). Quantification of band intensity was carried out using the Li-Cor Image Studio 3.1 software.

### 2.7. SIRT1 and SIRT3 Activity Assay

As SIRT4 activity assay was not commercially available, we determined enzyme activity of SIRT1 and SIRT3. Sirtuin deacetylase activity was measured using SIRT1 and SIRT3 Fluorometric Drug Discovery Kits (Flour de Lys, Enzo Life Sciences GmbH, Lörrach, Germany). On 96-well plates, we incubated 15 µL HEPES buffer containing 3750 cells with 15 µL of substrate mix, containing 0.1 mM Fluor de Lys© substrate and 5 mM NAD^+^ dissolved in 20 µL assay buffer. For the deacetylation reaction, the mixture was incubated for 15 min at 37 °C. The reaction was stopped by adding 50 µL of developer mix, containing 76% (v/v) assay buffer, 20% (v/v) Flour de Lys© Developer (Enzo Life Sciences GmbH, Lörrach, Germany) and 4% nicotinamide (50 mM). The plates were incubated for 45 min at 37 °C. In a fluorescence plate reader (excitation wavelength 360 nm, emission wavelength 460 nm), the activities were determined using an Infinite 200 microplate reader (Tecan Group Ltd., Männedorf, Switzerland). As measurements were performed under substrate saturation in vitro, the activities measured did not necessarily reflect in vivo activities, but rather reflected the enzyme capacities under optimal conditions in vitro.

### 2.8. Determination of NAD^+^

We took 250 µL of the HEPES-lysate used for the sirtuin deacetylase activity assay, precipitated the remaining protein by adding 900 µL 0.5% (w/v) sodium deoxycholate and 100 µL of 50% (w/v) trichloracetic acid (TCA), for 15 min, followed by centrifugation for 10 min at 13,000 × g and 4 °C.

A total of 20 µL of this protein-free supernatant lysate, 20 µL 100% ethanol, and 95 µL reaction mixture, containing 100 mM sodium pyrophosphate and 45 mM semicarbacide, were mixed in a 96-well reaction plate. By adding 15 µL alcohol dehydrogenase (10 U/mL), the oxidation of the alcohol under consumption of NAD^+^ was started.

The absorption at the wavelength of 340 nm was measured spectrophotometrically in an Infinite 200 microplate reader (Tecan, CH), at different time-points. The relative NAD^+^ values compared to the control cells kept under normoxic conditions were calculated by the mean differences between the initial and the final NADH-amount.

### 2.9. Statistical Methods

Statistical analyses were performed with GraphPad Prism 6.01 (GraphPad Software, San Diego, USA). In the graphs, data are presented as median with upper and lower quantile and 5% and 95% percentile. D’Agostino-Pearson was used for testing normality. Further statistics were performed using the Kruskal-Wallis test. Differences were regarded as significant at *p*-values of * *p* < 0.05; ** *p* < 0.01.

## 3. Results

**Figure 1:**
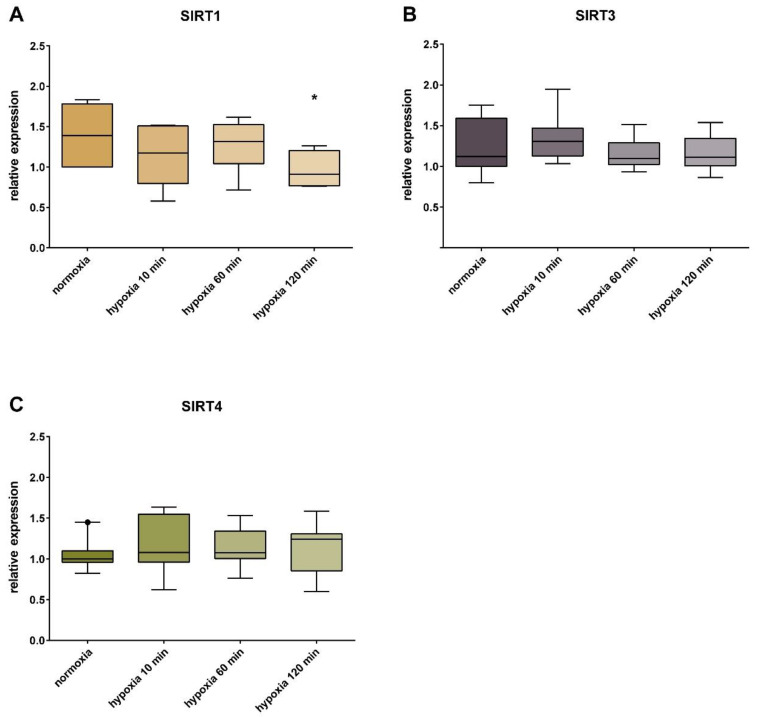
Relative gene expression of sirtuins in human umbilical vein endothelial cells (HUVECs). Expression of SIRT1 (**A**), SIRT3 (**B**), and SIRT4 (**C**) in HUVECs. Controls under the normoxic conditions were compared with cells incubated under hypoxia for 10, 60, and 120 min. Shown are the median, upper, and lower quartile, and the 5% and 95% percentile. Each group had a sample size of *n* = 3. Significance: * = *p* < 0.05.

We observed a significant reduction of SIRT1 at the transcript level, by 34% (± 0.22% SD), after hypoxia treatment with 2% O₂ for 120 min, compared to the cells incubated under normoxic conditions. No significant change in the transcript content of SIRT 3 and SIRT4 was detected (Figure 1).

**Figure 2:**
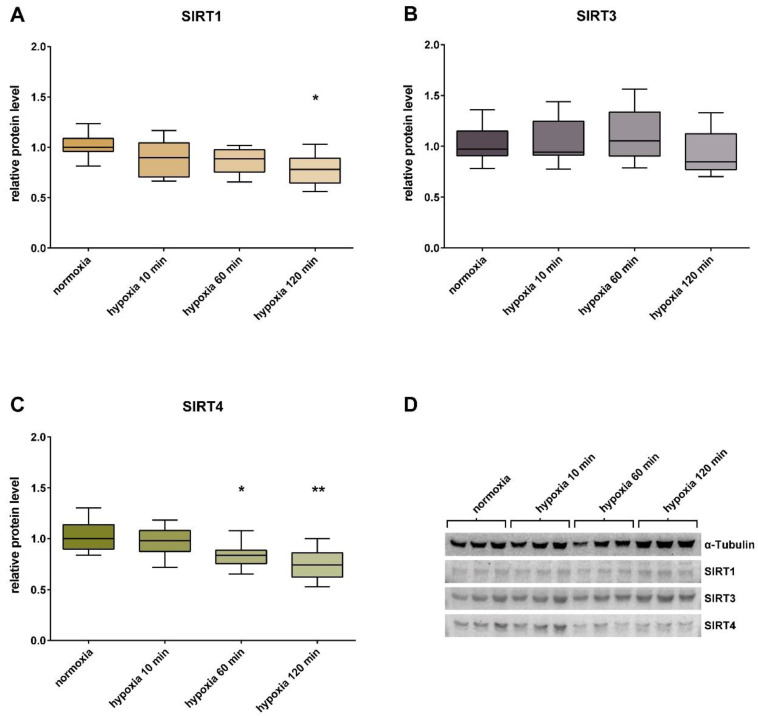
Western blot analysis and relative protein content of sirtuins in HUVECs. The relative protein content of SIRT1 (**A**), SIRT3 (**B**), and SIRT4 (**C**) in HUVECs. Controls under the normoxic conditions were compared with cells incubated under hypoxia for 10, 60, and 120 min. Shown are the median, upper, and lower quartile, and the 5% and 95% percentile. Each group had a sample size of n = 3. Significance: * *p* < 0.05 and ** *p* < 0.005. Representative Western blot analysis (**D**) of cells incubated under normoxia (21% O_2_) and hypoxia (2% O_2_) for 10, 60, and 120 min. α-Tubulin ~52kDa; SIRT1 ~110 kDa; SIRT3 ~44 kDa; SIRT4 ~39 kDa.

At protein level, SIRT1 decreased under hypoxic conditions. The relative protein content of SIRT1 showed a significant reduction by 22% (± 0.15% SD), after incubation for 120 min under 2% O₂. For SIRT4, a significant reduction of the relative protein content by 16% (± 0.12% SD) could be detected after 60 min of hypoxia treatment, and by 26% (± 0.15% SD) after 120 min; SIRT3 remained unchanged (Figure 2).

**Figure 3:**
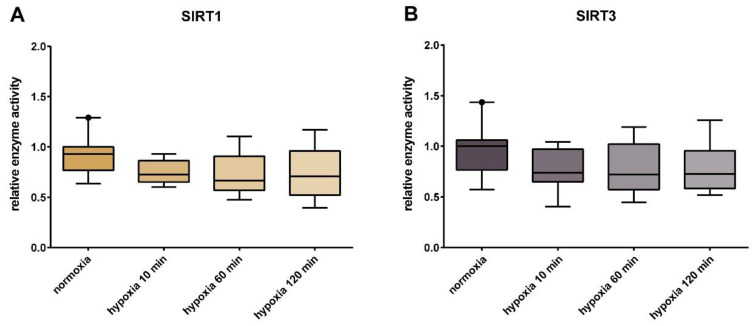
Relative sirtuin enzyme capacity in the HUVECs. Relative maximum activity of SIRT1 (**A**) and SIRT3 (**B**) in the HUVECs. Normoxic controls were compared with cells incubated under hypoxia for 10, 60, and 120 min. Shown are the median, upper, and lower quartile, and the 5% and 95% percentile. Each group had a sample size of *n* = 3. Normoxic SIRT1 enzyme capacity averaged 1.8 U/10,000 cells in the normoxic HUVECs. The normoxic enzyme activity of SIRT3, averaged 9.5 U/µg protein.

Sirtuin capacity levels (in vitro activity under substrate saturation and optimal pH) tended to decrease during short-term hypoxia treatment, but without a significant difference (Figure 3). Basal SIRT1 enzyme capacity (corresponding to 1.0) averaged 1.8 U/10,000 cells in the normoxic HUVECs. The basal enzyme activity of SIRT3 averaged 9.5 U/µg protein.

**Figure 4:**
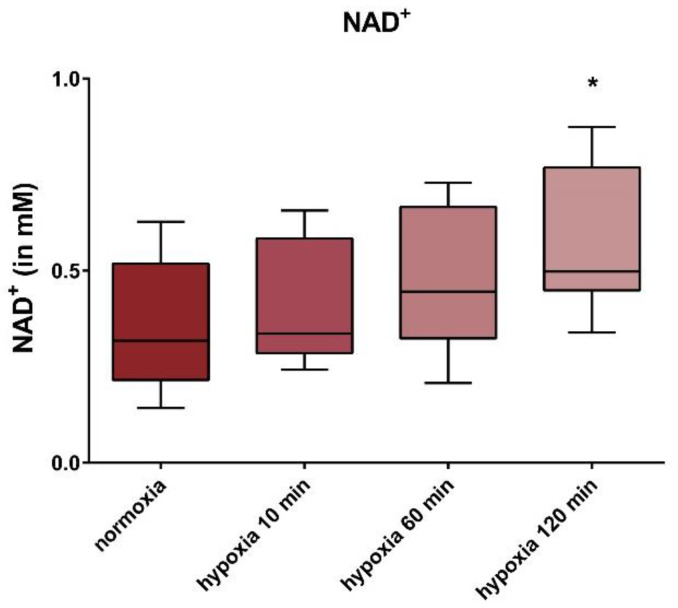
Intracellular NAD^+^ concentration in the HUVECs. The normoxic controls were compared with the cells incubated under hypoxia for 10, 60, and 120 min. The NAD^+^ concentration is presented in mM. Shown are the median, upper, and lower quartile, as well as the 5% and 95% percentile. Each group had a sample size of *n* = 3. Significance: * *p* < 0.05.

In all cell lines, we observed an upward trend of NAD^+^ levels, at incubation times of 10 min and 60 min under hypoxic conditions. A significant increase of 57% (± 0.19% SD) was observed after an incubation time of 120 min, under hypoxia (Figure 4).

## 4. Discussion

Cardiovascular diseases are often associated with endothelial dysfunction, such as impaired eNOS activity and diminished NO bioavailability. The pathophysiological processes underlying this phenomenon are incompletely understood [71,72]. Hypoxia might contribute to hampered endothelial function and downregulation of energy metabolism. Sirtuins are important regulatory elements of energy metabolism and antioxidative defense, using post-translational modifications [32,52,73,74]. Sirtuins were recently shown to regulate vascular and especially endothelial functions, under physiological conditions [1,2,3,4]. Dysregulation of sirtuins was observed under pathophysiological conditions like cardiovascular disease [20,21,22,23,24] and vascular aging, including atherosclerosis [1,25,26,27,28].

Cellular stress due to physical exercise can upregulate aerobic energy production and sirtuins, not only via post-translational modification but also indirectly via increased substrate (NAD^+^) saturation (e.g., [54], for a recent review see Das and Dabke 2020 [32], manuscript in press) in different tissues.

We hypothesized that acute, short-term hypoxia might trigger abnormal functions of sirtuins in endothelial cells, thus contributing to endothelial dysfunction. In the present study, we investigated the effects of short-term hypoxia on SIRT1, SIRT3, and SIRT4 in normal HUVECs. We observed a significant reduction of SIRT1 and SIRT4 protein levels, a downregulation of SIRT1 at the transcript level and an increase of NAD^+^ during hypoxia in HUVECs.

The relative gene expression of SIRT1 in HUVECs showed a significant reduction by 52% after 120 min of hypoxia, compared to normoxia. In line with our findings, reduced SIRT1 expression under hypoxia was previously demonstrated in renal and fibrosarcoma cells [52]. In a previous study, we observed decreased SIRT1 and SIRT4 transcript levels in fetal endothelial cells and HUVECs from pregnancies complicated by gestational diabetes, where a hypoxic component can also be postulated [75]. In the present study, the relative protein content of SIRT1 also decreased significantly by 22%, after 120 min of hypoxia treatment. It is noticeable that the reduction in protein content is not of the same magnitude as the reduction in relative gene expression, but is smaller. A possible explanation for this difference could be that short-term change in expression has little influence on the amount of protein, as sirtuin enzymes have a longer half-life than sirtuin mRNA. It is not clear how hypoxia reduces SIRT1 and SIRT4 in the protein and at the expression levels. Both sirtuins are metabolic sensors regulated by different metabolites. For SIRT1, modulation of expression was described via carboxyl terminus binding protein (CtBP) [52]. Furthermore, SIRT1 gene expression was regulated in hypoxia in a HIF-dependent manner [76]. The CREB (cAMP-responsive element protein-binding) protein was able to regulate SIRT1 expression in response to the NAD/NADH-levels [77]. Further experimentation is necessary to elucidate the underlying mechanism.

Under hypoxic conditions, the flux of the mitochondrial respiratory chain is switched off. For energy supply and maintenance of cellular functions and survival, the cells have to rely on less efficient anaerobic glycolysis. The respiratory chain is a considerable potential source of ROS. However, shutting down the respiratory chain during hypoxia also has a protective effect. SIRT1 was shown to boost antioxidative defense via PGC-1 alpha and the AMPK-SIRT1 pathway (for a review see Thirupathi et al. [78]). The downregulation of SIRT1 observed under hypoxia in our study hampered this protective effect.

Endothelial cells only have very few mitochondria and mainly generate their energy from glycolysis [24], nevertheless, the loss of mitochondrial integrity results in endothelial dysfunction and vascular disease [18]. Mitochondria have other functions beyond energy production in endothelial cells. Calcium homeostasis might be hampered, there is cross-talk of mitochondria with other cell organelles, and posttranslational modification of proteins like NOS might be triggered by mitochondrial alterations [19]. ROS might be important intermediates, however, based on the low number of mitochondria in HUVECs, the amount of ROS might not be high. Further experimentation is required to characterize these mitochondrial functions under hypoxic conditions.

The NAD^+^ levels increased under hypoxia, which might partly compensate the reduced SIRT1 activity.

SIRT1 was discussed to contribute to atherosclerosis [33,34]. Furthermore, SIRT1 was shown to activate the eNOS. SIRT1-downregulation occurring during acute hypoxia might hamper NO production [79], thus, compromising blood flow. The use of sirtuin activators (e.g., resveratrol) might have a protective effect [19,80,81]. Being posttranslational modifiers, they might act quickly and maintain blood flow. Exercise training during cardiovascular rehabilitation might also increase sirtuin activities [16,82], which might be beneficial in future hypoxic episodes. However, further experimentation is necessary to study the effect of SIRT1 activation in vivo, which is expected to (partly) compensate the downregulation of the SIRT1 capacity.

We observed elevation of NAD^+^ levels under hypoxic conditions (120 min), in our study. This might presumably enhance sirtuin activity via increased substrate provision in the intact cell. However, we would expect a decrease in NAD^+^ levels under hypoxia, based on the accumulation of NADH under hypoxia with blocked respiratory chain, and a subsequent shift of NAD^+^ to NADH. Indeed, a decrease in NAD^+^ concentrations was shown in other cells under hypoxia for 8 h [52]. One possible explanation for the significant increase in NAD^+^ concentrations under hypoxia, in our study in HUVECs, could be the significantly decreased SIRT1 expression and the SIRT1 and SIRT4 protein levels. A reduced turnover of the essential cofactor due to the reduced sirtuin content could thus contribute to an increase in the NAD^+^ content. An increased biosynthesis of NAD^+^ from nicotinamide or de novo synthesis of NAD^+^ from tryptophan might also involved. We suppose regulation of mitochondrial transcripts via a second messenger. It is known that some sirtuins are under the transcriptional control of FOXO transcription factors, which regulate the stress response [83,84]. Further experimentation is necessary to elucidate the mechanisms that lead to NAD^+^ elevation under hypoxic conditions. Downregulation of SIRT1 and NAD^+^ was observed during aging [1,22,25,26,27,28], due to age-related atherosclerosis, presumably resulting from impaired eNOS activity [33,34].

Many different proteins that play a crucial role in controlling ROS production and ROS elimination (e.g., via SOD2, FOXO3, IDH2, etc.) are deacetylated by SIRT3. In numerous studies, it was shown that increased ROS due to long-term hypoxia can induce SIRT3 expression, protein level and enzyme activity, thus initiating a program to eliminate ROS [51,61,85]. In our study, we did not observe alterations of SIRT3 under hypoxia. Nevertheless, the elevated NAD^+^ levels might enhance SIRT3 activity in vivo.

In our study, we observed a significant reduction in SIRT4 protein levels after 60 and 120 min of hypoxia. SIRT4 regulates ATP homeostasis via ANT2 (adenine nucleotide translocase 2) [63]. When SIRT4 functions normally, ANT2 is deacetylated and acts as a transport protein of ADP/ATP across the inner mitochondrial membrane [63]. However, in the acetylated state, the mitochondrial decoupling function of ANT2 is activated. The reduced SIRT4 protein content under hypoxia might lead to decoupling, due to the reduced posttranslational deacetylation of ANT2. Under hypoxia, the mitochondrial F_0_F_1_-ATP synthase functions as ATPase [86,87]. Decoupling leads to a reduction of the proton gradient, across the inner mitochondrial membrane. This impairs the function of the F_0_F_1_-ATPase by the binding of an inhibitor protein, thus limiting ATP hydrolysis [86,87]. Furthermore, ANT2 is able to transport glycolytic ATP from the cytosol to the mitochondrial matrix [88], where it is hydrolyzed by the mitochondrial F_0_F_1_-ATP synthase, under hypoxic conditions [86,87].

In the case of SIRT4 reduction, mitochondrial fatty acid uptake is subsequently increased. Under physiological conditions, malonyl-CoA has an inhibitory effect on CPT1. Reduced SIRT4 leads to hyperacetylation and activation of the malonyl CoA-decarboxylase, with a subsequent drop in the malonyl-CoA levels and increased fatty acid uptake to the mitochondrial matrix [64]. The increased mitochondrial fatty acid transport capacity could be interpreted as a ‘predatory’ mechanism to quickly replenish depleted ATP stores during re-oxygenation, thus, improving blood flow. This adaptation of the energy supply seems to make sense, because PDH is inactivated via ROS accumulation under hypoxia, which prevents the entry of glucose into the citrate cycle, and thus reduces energy production from glycolysis [89]. Mathias et al. showed that SIRT4 has lipidase activity and can also inactivate PDH [90]. Thus, increased PDH activity rates can be expected in the context of a SIRT4 downregulation and the release of the negative regulation of PDH. Consequently, more acetyl-CoA is produced and glycolysis is linked to the citrate cycle. This ensures an alternative energy supply for the cell, once re-oxygenation starts. In the SIRT4 knockout mouse model, this metabolic adaptation was already demonstrated. Interestingly, in this case, both the mitochondrial and the cardiac ROS levels were reduced [91]. In our study, ROS levels were not measured.

SIRT4 was shown to prevent endothelial dysfunction and to repress inflammatory processes in response to cellular stress [24,92,93]. Downregulation of SIRT4 enhances pro-inflammatory cytokines and hence contributes to vascular damage [24].

The limitations of our study were as follows. The experiments were carried out in vitro. Effects in HUVECs might be different from arterial endothelial cells that are exposed to higher blood pressure. Aging endothelial cells might also be different from HUVECs in terms of sirtuin response to hypoxia. Another limitation is that ROS levels were not measured. In our study, we chose short-term hypoxia to simulate conditions often occurring under pathological conditions. It seems that remarkable effects of hypoxia on sirtuins in HUVECs can already be observed in the first 2 h. Our conclusions reflect acute changes and we can only speculate on long-term hypoxia. Yet, studies from our and other groups in HUVECs and other cells and animals, strongly indicate manifest effects of short-term hypoxia on several sirtuins, including SIRT1 [52,75]

Our in vitro findings might have implications for clinical medicine. Modern pathophysiological concepts regard atherosclerosis as an immunometabolic disease, with sirtuins playing an important role [21,22,23,24]. SIRT1, which decreases with age, is supposed to contribute to age-related endothelial dysfunction by compromising eNOS [25]. According to our results, acute short-term hypoxia might play an important additional role in the dysregulation of sirtuins, namely of SIRT1 and SIRT4, in cardiovascular disorders. Further experimentation, not limited to cell models but also examining whole organs in animals are necessary. This will allow us to induce ischemia, as opposed to hypoxia.

## 5. A Clinical Medical Outlook

Under acute hypoxia, SIRT1 and SIRT4 were downregulated in healthy HUVECs from uncomplicated pregnancies, whereas intracellular NAD^+^ levels were increased. SIRT1 and SIRT4 downregulation might result in increased oxidative stress and inflammation, and two atherogenic and aging factors. Activators of sirtuins were shown in animals and humans to reverse vascular dysfunction, if started early. Resveratrol administration might increase NO production and decrease oxidative stress [4]. NAD^+^ boosters have a beneficial effect in cardiovascular disorders [1,21,22,23,24,26,27]. Activators of sirtuins and NAD^+^ boosters might be useful in preventing exacerbation of atherosclerosis during acute hypoxic events, such as stroke and cardiac infarction. Such drugs might also be beneficial in other hypoxic conditions, such as organ transplantation, perinatal hypoxia, and gestational diabetes mellitus [75]. Further studies are necessary to test these compounds in vivo.
